# The 100 most cited articles about orofacial trauma in children and adolescents: bibliometric analysis

**DOI:** 10.1590/1807-3107bor-2024.vol38.0080

**Published:** 2024-09-02

**Authors:** Priscila Seixas MOURÃO, Izabella Barbosa FERNANDES, Gabrielly Fernandes MACHADO, Rodrigo GALO, Luna Chagas CLEMENTINO, Paulo Antônio MARTINS-JÚNIOR, Maria Letícia RAMOS-JORGE

**Affiliations:** (a) Universidade Federal dos Vales do Jequitinhonha e Mucuri – UFVJM, Department of Pediatric Dentistry and Orthodontics, Diamantina, Brazil. Diamantina, MG, Brazil.; (b) Universidade Federal de Minas Gerais – UFMG, School of Dentistry, Department of Pediatric Dentistry, Belo Horizonte, MG, Brazil.; (c) Universidade de São Paulo – USP, Dental School of Ribeirão Preto,Department of Prosthodontics and Dental Materials, Ribeirão Preto, SP, Brazil.

**Keywords:** Adolescent, Bibliometrics, Child, Traumatology

## Abstract

This bibliometric study aimed to identify and analyze the 100 most cited articles about orofacial trauma in children and adolescents. The search was conducted in the Web of Science Core Collection (WoS-CC) using a combined search strategy. Two researchers collected the following data from each article: year of publication, country, journal, number and density of citations, author, institutions, study design, type of trauma, and keywords. The VOSviewer and SPSS version 22.0 softwares were used for data analyses. The articles were published from 1968 to 2012 and the number of citations ranged from 49 to 176. Europe was the continent with most articles (40 articles; 3,408 citations). Brazil was the country that made the largest contribution (20 articles; 1,741 citations) and the Universidade do Sul de Santa Catarina (Brazil) was the institution with most articles (5 articles; 492 citations). Marcenes W was the most productive author (8 articles; 968 citations). The cross-sectional study design was the most common (50 articles; 3,978 citations). The most frequent field was epidemiology (73 articles; 5,971 citations). The most widely used criteria for trauma diagnosis were the Andreasen (18 articles; 1,505 citations) and Le Fort (3 articles; 260 citations). Strong positive correlations were found in the number of citations between WoS-CC and Google Scholar (r = 0.929; p < 0.001), WoS-CC and Scopus (r = 0.976; p < 0.001), and Google Scholar and Scopus (r = 0.903; p < 0.001). The 100 most cited articles about orofacial trauma in children and adolescents were mainly cross-sectional studies published by Brazilian authors in epidemiology using Andreasen criteria. Dental Traumatology was the journal with the largest contribution.

## Introduction

Orofacial trauma can affect the tooth enamel, dental pulp, soft tissues, and hard tissues.^1^ The prevalence of traumatic dental injury is high among children and adolescents, with rates of 22.7% in primary dentition and 15.2% in permanent dentition, and the prevalence of maxillofacial trauma ranges from 2.8 to 89.7%.^2-4^

Individuals who suffer orofacial trauma may need less invasive treatment, such as monitoring, incisal adjustments or the use of a retainer, or more invasive treatment, such as prosthetic restorations, endodontic treatment or surgical procedures with or without general anesthesia.^5,6^ There is also evidence that trauma can exert a physical impact and can influence the quality of life of affected individuals both directly and indirectly by generating economic impact.^7,8^

Bibliometric analyses are scientific methods that enable the identification of articles with greater impact in both the research and clinical community, using metrics such as the number of citations that these studies receive over time.^9,10^ The quantitative analysis of these articles can be used to follow the development of particular fields of research.^11^ Bibliometric studies enable clinicians to identify the main studies on a given topic. Moreover, bibliometrics can reveal research trends, main authors, and journals and countries with the most publication activity, as well as emerging and promising fields of research,^9^thereby inspiring the development of new studies.^12^ Although bibliometric studies on orofacial trauma have been published in the literature,^13-16^ no studies have addressed the subject with a focus on children and adolescents, despite the high prevalence of orofacial trauma in this age group.

The aim of the present bibliometric study was to identify and analyze the 100 most cited articles about orofacial trauma in children and adolescents and help inform researchers and clinicians with regards to current and future research trends.

## Methods

A bibliometric study was conducted on May 20^th^, 2021 to identify and analyze the most cited articles about orofacial trauma in children and adolescents. The search was performed in the “Dentistry, Oral Surgery and Medicine” category of the Web of Science Core Collection (WoS-CC) database using a combination of MeSH terms and keywords (Box ).

**Box:** Search strategy used in the Web of Science Core Collection.


TS=(child OR children OR childhood OR child, preschool OR “preschool child” OR “preschool children” OR infant OR infants OR toddler OR toddlers OR preschool OR preschoolers OR schoolchild OR “school child” OR schoolchildren OR “school children” OR kid OR kids OR newborn OR newborns OR youth OR youths OR pediatric OR pediatrics OR paediatric OR paediatrics OR pedodontic OR pedodontics OR adolescent OR adolescents OR adolescence OR teen OR teens OR teenager OR teenagers OR offspring OR student OR students) AND TS=(tooth OR teeth OR dent* OR odont* OR mouth OR oral OR facial OR maxill* OR mandib* OR alveol* OR periodont* OR root*) AND TS=(trauma* OR injur* OR intrud* OR extrusi* OR extrud* OR avuls* OR ex$articulation* OR luxat* OR fractur* OR fragment* OR lacerat* OR subluxat* OR concus* OR re$plant* OR re$implant* OR crack* OR mobil*) NOT TS=(adult OR adults OR elderly)

A list of articles was organized by the number of citations in decreasing order. On the same day, two researchers simultaneously examined the title and abstract of all articles identified based on the eligibility criteria. The inclusion criteria were: articles involving any aspect of orofacial trauma in children and adolescents. The exclusion criteria were: editorials, conference papers, guidelines, studies including adults in the sample, studies in which the age of the participants was not informed, and studies that addressed conditions other than orofacial trauma, such as dental caries. The selection stopped at the hundredth most cited article and the full texts were obtained for analysis. Those articles in which a clear decision about inclusion/exclusion was not possible from the title/abstract evaluation also underwent full-text analysis. Disagreements about the inclusion/exclusion of articles were resolved by consensus with a third researcher, who was also present at the time of data collection.

The position of the articles on the list was based on the number of citations in WoS-CC. On the same day, a cross-match was performed with the number of citations in Scopus and Google Scholar for citation comparison. If there was a tie in the number of citations, the position of the articles on the list was determined by the citation density (number of citations per year) according to WoS-CC, followed by the number of citations in Scopus.

An electronic spreadsheet (Microsoft Excel^®^, Windows 10 version) was exported from the WoS-CC containing automatically data extracted from each article, such as number of citations, authors’ names, corresponding author’s address, journal title, keywords, and year of publication. The following data were manually extracted from each article: citation ratio, study design, type of orofacial trauma, age range of the sample, type of dentition, institution, country and continent (based on the affiliation of the corresponding author), and criteria used for the diagnosis and definition of the types of orofacial trauma.

The articles were classified as review (systematic or non-systematic), observational studies (cross-sectional, case-control, cohort, or ecological), interventional (clinical studies), and case reports/case series based on the Cochrane glossary.^17^ The fields were subdivided into epidemiology, prevention, treatment, and prognosis. Studies with 100 or more citations were considered highly cited.^18,19^

The VOSviewer software (Centre for Science and Technology Studies, Leiden University) was used to create the bibliometric networks.^20^ Collaborative density maps were created for co-authorship and keywords and items were linked considering the number of articles with joint authorship. Each point on the density map has a color corresponding to the density of the items on that point. The greater the number of items close to a point and the greater the weight of these items, the closer the color is to red, whereas the fewer number of items close to a point and the lower the weight of the neighboring items, the closer to color is to blue.

Data analysis was performed using the Statistical Package for the Social Sciences (SPSS, version 24.0 for Windows; IBM Corp). The Kolmogorov-Smirnov was used to assess the normality of data distribution. As a non-normal distribution was revealed, Spearman’s correlation test was used. The significance level was set at 5% (p < 0.05).

## Results

The search in WoS-CC retrieved 3,223 articles. After the organization of the articles in decreasing order number of citations, 144 articles were excluded for not addressing the topic of interest and 53 others were excluded mainly for the following reasons: including adults in the sample, not reporting the age of the participants, addressing conditions other than orofacial trauma such as dental caries, editorials, conference articles, and guidelines. The list of the 100 most cited articles about orofacial trauma in children and adolescents is displayed in Table 1.

**Table 1:** The top 100 most-cited articles on orofacial trauma in children and adolescents.

Rank	Articles	Number of citations (citation densityª)		

		WoS Core	Google Scholar	Scopus
1	Posnick JC, Wells M, Pron GE. Pediatric facial fractures: evolving patterns of treatment. J Oral Maxillofac Surg. 1993 Aug;51(8):836-44; discussion 844-5. https://doi.org/10.1016/s0278-2391(10)80098-9	176 (6.52)	349 (12.93)	204 (7.56)
2	Glendor U, Halling A, Andersson L, Eilert-Petersson E. Incidence of traumatic tooth injuries in children and adolescents in the county of Västmanland, Sweden. Swed Dent J. 1996;20(1-2):15-28.	168 (7.00)	335 (13.96)	179 (7.46)
3	Marcenes W, al Beiruti N, Tayfour D, Issa S. Epidemiology of traumatic injuries to the permanent incisors of 9-12-year-old schoolchildren in Damascus, Syria. Endod Dent Traumatol. 1999 Jun;15(3):117-23.https://doi.org/10.1111/j.1600-9657.1999.tb00767.x	153 (7.29)	390 (18.57)	175 (8.33)
4	Hamilton FA, Hill FJ, Holloway PJ. An investigation of dento-alveolar trauma and its treatment in an adolescent population. Part 1: The prevalence and incidence of injuries and the extent and adequacy of treatment received. Br Dent J. 1997 Feb 8;182(3):91-5. https://doi.org/10.1038/sj.bdj.4809313	142 (6.17)	358 (15.57)	160 (6.96)
5	Ravn JJ. Dental injuries in Copenhagen schoolchildren, school years 1967-1972. Community Dent Oral Epidemiol. 1974;2(5):231-45. https://doi.org/10.1111/j.1600-0528.1974.tb01658.x	140 (3.04)	267 (5.80)	152 (3.30)
6	Cortes MI, Marcenes W, Sheiham A. Prevalence and correlates of traumatic injuries to the permanent teeth of schoolchildren aged 9-14 years in Belo Horizonte, Brazil. Dent Traumatol. 2001 Feb;17(1):22-6. https://doi.org/10.1034/j.1600-9657.2001.170105.x	135 (7.11)	296 (15.58)	155 (8.16)
7	Marcenes W, Alessi ON, Traebert J. Causes and prevalence of traumatic injuries to the permanent incisors of school children aged 12 years in Jaragua do Sul, Brazil. Int Dent J. 2000 Apr;50(2):87-92. https://doi.org/10.1002/j.1875-595x.2000.tb00804.x	135 (6.75)	300 (15.00)	153 (7.65)
8	Nguyen QV, Bezemer PD, Habets L, Prahl-Andersen B. A systematic review of the relationship between overjet size and traumatic dental injuries. Eur J Orthod. 1999 Oct;21(5):503-15. https://doi.org/10.1093/ejo/21.5.503	135 (6.43)	281 (13.38)	158 (7.52)
9	Flores MT. Traumatic injuries in the primary dentition. Dent Traumatol. 2002 Dec;18(6):287-98. https://doi.org/10.1034/j.1600-9657.2002.00153.x	126 (7.00)	322 (17.89)	153 (8.50)
10	Borum MK, Andreasen JO. Sequelae of trauma to primary maxillary incisors. I. Complications in the primary dentition. Endod Dent Traumatol. 1998 Feb;14(1):31-44. https://doi.org/10.1111/j.1600-9657.1998.tb00806.x	123 (5.59)	302 (13.73)	146 (6.54)
11	Marcenes W, Murray S. Social deprivation and traumatic dental injuries among 14-year-old schoolchildren in Newham, London. Dent Traumatol. 2001 Feb;17(1):17-21. https://doi.org/10.1034/j.1600-9657.2001.170104.x	119 (6.26)	216 (11.37)	116 (6.11)
12	Kramer PF, Zembruski C, Ferreira SH, Feldens CA. Traumatic dental injuries in Brazilian preschool children. Dent Traumatol. 2003 Dec;19(6):299-303. https://doi.org/10.1046/j.1600-9657.2003.00203.x	116 (6.82)	262 (15.41)	125 (7.35)
13	Traebert J, Peres MA, Blank V, Böell Rda S, Pietruza JA. Prevalence of traumatic dental injury and associated factors among 12-year-old school children in Florianópolis, Brazil. Dent Traumatol. 2003 Feb;19(1):15-8. https://doi.org/10.1034/j.1600-9657.2003.00138.x	113 (6.65)	277 (16.29)	125 (7.35)
14	Traebert J, Peres MA, Blank V, Böell Rda S, Pietruza JA. Prevalence of traumatic dental injury and associated factors among 12-year-old school children in Florianópolis, Brazil. Dent Traumatol. 2003 Feb;19(1):15-8. https://doi.org/10.1034/j.1600-9657.2003.00138.x	112 (4.67)	270 (11.25)	122 (5.08)
15	Soriano EP, Caldas Ade F Jr, Diniz De Carvalho MV, Amorim Filho Hde A. Prevalence and risk factors related to traumatic dental injuries in Brazilian schoolchildren. Dent Traumatol. 2007 Aug;23(4):232-40. https://doi.org/10.1111/j.1600-9657.2005.00426.x	108 (8.31)	256 (19.69)	127 (9.77)
16	Andreasen JO, Andreasen FM, Mejàre I, Cvek M. Healing of 400 intra-alveolar root fractures. 1. Effect of pre-injury and injury factors such as sex, age, stage of root development, fracture type, location of fracture and severity of dislocation. Dent Traumatol. 2004 Aug;20(4):192-202. https://doi.org/10.1111/j.1600-9657.2004.00279.x	105 (6.56)	242 (15.13)	113 (7.06)
17	Skaare AB, Jacobsen I. Dental injuries in Norwegians aged 7-18 years. Dent Traumatol. 2003 Apr;19(2):67-71. https://doi.org/10.1034/j.1600-9657.2003.00133.x	102 (6.00)	209 (12.29)	114 (6.71)
18	Pohl Y, Filippi A, Kirschner H. Results after replantation of avulsed permanent teeth. II. Periodontal healing and the role of physiologic storage and antiresorptive-regenerative therapy. Dent Traumatol. 2005 Apr;21(2):93-101. https://doi.org/10.1111/j.1600-9657.2004.00298.x	101 (6.73)	225 (15.00)	106 (7.07)
19	Borssén E, Holm AK. Traumatic dental injuries in a cohort of 16-year-olds in northern Sweden. Endod Dent Traumatol. 1997 Dec;13(6):276-80. https://doi.org/10.1111/j.1600-9657.1997.tb00055.x	101 (4.39)	250 (10.87)	118 (5.13)
20	Burden DJ. An investigation of the association between overjet size, lip coverage, and traumatic injury to maxillary incisors. Eur J Orthod. 1995 Dec;17(6):513-7. https://doi.org/10.1093/ejo/17.6.513	100 (4.00)	197 (7.88)	107 (4.28)
21	Rocha MJ, Cardoso M. Traumatized permanent teeth in Brazilian children assisted at the Federal University of Santa Catarina, Brazil. Dent Traumatol. 2001 Dec;17(6):245-9. https://doi.org/10.1034/j.1600-9657.2001.170601.x	99 (5.21)	228 (12.00)	111 (5.84)
22	Eggensperger Wymann NM, Hölzle A, Zachariou Z, Iizuka T. Pediatric craniofacial trauma. J Oral Maxillofac Surg. 2008 Jan;66(1):58-64. https://doi.org/10.1016/j.joms.2007.04.023	96 (8.00)	176 (14.67)	98 (8.17)
23	Chan AW, Wong TK, Cheung GS. Lay knowledge of physical education teachers about the emergency management of dental trauma in Hong Kong. Dent Traumatol. 2001 Apr;17(2):77-85. https://doi.org/10.1034/j.1600-9657.2001.017002077.x	94 (4.95)	279 (14.68)	107 (5.63)
24	Traebert J, Bittencourt DD, Peres KG, Peres MA, de Lacerda JT, Marcenes W. Aetiology and rates of treatment of traumatic dental injuries among 12-year-old school children in a town in southern Brazil. Dent Traumatol. 2006 Aug;22(4):173-8.https://doi.org/10.1111/j.1600-9657.2006.00359.x	92 (6.57)	191 (13.64)	100 (7.14)
25	Oliveira LB, Marcenes W, Ardenghi TM, Sheiham A, Bönecker M. Traumatic dental injuries and associated factors among Brazilian preschool children. Dent Traumatol. 2007 Apr;23(2):76-81. https://doi.org/10.1111/j.1600-9657.2005.00413.x	91 (7.00)	187 (14.38)	96 (7.38)
26	Nicolau B, Marcenes W, Sheiham A. Prevalence, causes and correlates of traumatic dental injuries among 13-year-olds in Brazil. Dent Traumatol. 2001 Oct;17(5):213-7. https://doi.org/10.1034/j.1600-9657.2001.170505.x	91 (4.79)	214 (11.26)	100 (5.26)
27	Forsberg CM, Tedestam G. Etiological and predisposing factors related to traumatic injuries to permanent teeth. Swed Dent J. 1993;17(5):183-90.	90 (3.33)	198 (7.33)	94 (3.48)
28	Forsberg CM, Tedestam G. Traumatic injuries to teeth in Swedish children living in an urban area. Swed Dent J. 1990;14(3):115-22.	90 (3.00)	193 (6.43)	104 (3.47)
29	Kaban LB. Diagnosis and treatment of fractures of the facial bones in children 1943-1993. J Oral Maxillofac Surg. 1993 Jul;51(7):722-9. https://doi.org/10.1016/s0278-2391(10)80409-4	89 (3.30)	205 (7.59)	116 (4.30)
30	Becker DB, Needleman HL, Kotelchuck M. Child abuse and dentistry: orofacial trauma and its recognition by dentists. J Am Dent Assoc. 1978 Jul;97(1):24-8. https://doi.org/10.14219/jada.archive.1978.0447	88 (2.10)	231 (5.50)	112 (2.67)
31	Hovinga J, Boering G, Stegenga B. Long-term results of nonsurgical management of condylar fractures in children. Int J Oral Maxillofac Surg. 1999 Dec;28(6):429-40.	86 (4.10)	147 (7.00)	84 (4.00)
32	Cvek M, Andreasen JO, Borum MK. Healing of 208 intra-alveolar root fractures in patients aged 7-17 years. Dent Traumatol. 2001 Apr;17(2):53-62. https://doi.org/10.1034/j.1600-9657.2001.017002053.x	84 (4.42)	179 (9.42)	100 (5.26)
33	von Arx T. Developmental disturbances of permanent teeth following trauma to the primary dentition. Aust Dent J. 1993 Feb;38(1):1-10. https://doi.org/10.1111/j.1834-7819.1993.tb05444.x	82 (3.04)	212 (7.85)	108 (4.00)
34	Rowe NL. Fractures of the facial skeleton in children. J Oral Surg. 1968 Aug;26(8):505-15.	81 (1.56)	174 (3.35)	98 (1.88)
35	Hamilton FA, Hill FJ, Mackie IC. Investigation of lay knowledge of the management of avulsed permanent incisors. Endod Dent Traumatol. 1997 Feb;13(1):19-23. https://doi.org/10.1111/j.1600-9657.1997.tb00004.x	80 (3.48)	169 (7.35)	92 (4.00)
36	Marcenes W, Zabot NE, Traebert J. Socio-economic correlates of traumatic injuries to the permanent incisors in schoolchildren aged 12 years in Blumenau, Brazil. Dent Traumatol. 2001 Oct;17(5):222-6. https://doi.org/10.1034/j.1600-9657.2001.170507.x	79 (4.16)	226 (11.89)	97 (5.11)
37	Barrett EJ, Kenny DJ. Survival of avulsed permanent maxillary incisors in children following delayed replantation. Endod Dent Traumatol. 1997 Dec;13(6):269-75. https://doi.org/10.1111/j.1600-9657.1997.tb00054.x	78 (3.39)	139 (6.04)	85 (3.70)
38	Altay N, Güngör HC. A retrospective study of dento-alveolar injuries of children in Ankara, Turkey. Dent Traumatol. 2001 Oct;17(5):201-4. https://doi.org/10.1034/j.1600-9657.2001.170502.x	77 (4.05)	168 (8.84)	88 (4.63)
39	Cunha RF, Pugliesi DM, de Mello Vieira AE. Oral trauma in Brazilian patients aged 0-3 years. Dent Traumatol. 2001 Oct;17(5):210-2. https://doi.org/10.1034/j.1600-9657.2001.170504.x	77 (4.05)	178 (9.37)	85 (4.47)
40	Fakhruddin KS, Lawrence HP, Kenny DJ, Locker D. Impact of treated and untreated dental injuries on the quality of life of Ontario school children. Dent Traumatol. 2008 Jun;24(3):309-13. https://doi.org/10.1111/j.1600-9657.2007.00547.x	75 (6.25)	148 (12.33)	81 (6.75)
41	Tapias MA, Jiménez-García R, Lamas F, Gil AA. Prevalence of traumatic crown fractures to permanent incisors in a childhood population: Móstoles, Spain. Dent Traumatol. 2003 Jun;19(3):119-22. https://doi.org/10.1034/j.1600-9657.2003.00141.x	75 (4.41)	183 (10.76)	82 (4.82)
42	Bamjee Y, Lownie JF, Cleaton-Jones PE, Lownie MA. Maxillofacial injuries in a group of South Africans under 18 years of age. Br J Oral Maxillofac Surg. 1996 Aug;34(4):298-302. https://doi.org/10.1016/s0266-4356(96)90006-6	75 (3.13)	177 (7.38)	83 (3.46)
43	Rajab LD. Traumatic dental injuries in children presenting for treatment at the Department of Pediatric Dentistry, Faculty of Dentistry, University of Jordan, 1997-2000. Dent Traumatol. 2003 Feb;19(1):6-11. https://doi.org/10.1034/j.1600-9657.2003.00131.x	74 (4.35)	220 (12.94)	83 (4.88)
44	Robson F, Ramos-Jorge ML, Bendo CB, Vale MP, Paiva SM, Pordeus IA. Prevalence and determining factors of traumatic injuries to primary teeth in preschool children. Dent Traumatol. 2009 Feb;25(1):118-22. https://doi.org/10.1111/j.1600-9657.2008.00725.x	73 (6.64)	136 (12.36)	73 (6.64)
45	Nicolau B, Marcenes W, Sheiham A. The relationship between traumatic dental injuries and adolescents’ development along the life course. Community Dent Oral Epidemiol. 2003 Aug;31(4):306-13. https://doi.org/10.1034/j.1600-0528.2003.t01-1-00019.x	73 (4.29)	118 (6.94)	78 (4.59)
46	Diab M, elBadrawy HE. Intrusion injuries of primary incisors. Part III: Effects on the permanent successors. Quintessence Int. 2000 Jun;31(6):377-84.	73 (3.65)	167 (8.35)	88 (4.40)
47	Sandalli N, Cildir S, Guler N. Clinical investigation of traumatic injuries in Yeditepe University, Turkey during the last 3 years. Dent Traumatol. 2005 Aug;21(4):188-94. https://doi.org/10.1111/j.1600-9657.2005.00309.x	72 (4.80)	203 (13.53)	77 (5.13)
48	Sae-Lim V, Lim LP. Dental trauma management awareness of Singapore pre-school teachers. Dent Traumatol. 2001 Apr;17(2):71-6. https://doi.org/10.1034/j.1600-9657.2001.017002071.x	72 (3.79)	193 (10.16)	85 (4.47)
49	Petrovic B, Marković D, Peric T, Blagojevic D. Factors related to treatment and outcomes of avulsed teeth. Dent Traumatol. 2010 Feb;26(1):52-9. https://doi.org/10.1111/j.1600-9657.2009.00836.x	71 (7.10)	195 (19.50)	83 (8.30)
50	Granville-Garcia AF, de Menezes VA, de Lira PI. Dental trauma and associated factors in Brazilian preschoolers. Dent Traumatol. 2006 Dec;22(6):318-22. https://doi.org/10.1111/j.1600-9657.2005.00390.x	71 (5.07)	140 (10.00)	76 (5.43)
51	Strobl H, Emshoff R, Röthler G. Conservative treatment of unilateral condylar fractures in children: a long-term clinical and radiologic follow-up of 55 patients. Int J Oral Maxillofac Surg. 1999 Apr;28(2):95-8.	71 (3.38)	134 (6.38)	70 (3.33)
52	Ramos-Jorge ML, Bosco VL, Peres MA, Nunes AC. The impact of treatment of dental trauma on the quality of life of adolescents - a case-control study in southern Brazil. Dent Traumatol. 2007 Apr;23(2):114-9. https://doi.org/10.1111/j.1600-9657.2005.00409.x	70 (5.38)	163 (12.54)	67 (5.15)
53	Reis A, Loguercio AD, Kraul A, Matson E. Reattachment of fractured teeth: a review of literature regarding techniques and materials. Oper Dent. 2004 Mar-Apr;29(2):226-33.	70 (4.38)	268 (16.75)	92 (5.75)
54	Al-Majed I, Murray JJ, Maguire A. Prevalence of dental trauma in 5-6- and 12-14-year-old boys in Riyadh, Saudi Arabia. Dent Traumatol. 2001 Aug;17(4):153-8. https://doi.org/10.1034/j.1600-9657.2001.170403.x	70 (3.68)	143 (7.53)	75 (3.95)
55	Bauss O, Röhling J, Schwestka-Polly R. Prevalence of traumatic injuries to the permanent incisors in candidates for orthodontic treatment. Dent Traumatol. 2004 Apr;20(2):61-6. https://doi.org/10.1111/j.1600-4469.2004.00230.x	68 (4.25)	141 (8.81)	77 (4.81)
56	Kargul B, Cağlar E, Tanboga I. Dental trauma in Turkish children, Istanbul. Dent Traumatol. 2003 Apr;19(2):72-5. https://doi.org/10.1034/j.1600-9657.2003.00091.x	68 (4.00)	161 (9.47)	69 (4.06)
57	Raphael SL, Gregory PJ. Parental awareness of the emergency management of avulsed teeth in children. Aust Dent J. 1990 Apr;35(2):130-3. https://doi.org/10.1111/j.1834-7819.1990.tb05878.x	68 (2.27)	159 (5.30)	83 (2.77)
58	Jorge KO, Moysés SJ, Ferreira e Ferreira E, Ramos-Jorge ML, de Araújo Zarzar PM. Prevalence and factors associated to dental trauma in infants 1-3 years of age. Dent Traumatol. 2009 Apr;25(2):185-9. https://doi.org/10.1111/j.1600-9657.2008.00730.x	67 (6.09)	134 (12.18)	63 (5.73)
59	Humphrey JM, Kenny DJ, Barrett EJ. Clinical outcomes for permanent incisor luxations in a pediatric population. I. Intrusions. Dent Traumatol. 2003 Oct;19(5):266-73. https://doi.org/10.1034/j.1600-9657.2003.00207.x	67 (3.94)	137 (8.06)	76 (4.47)
60	Malikaew P, Watt RG, Sheiham A. Prevalence and factors associated with traumatic dental injuries (TDI) to anterior teeth of 11-13 year old Thai children. Community Dent Health. 2006 Dec;23(4):222-7.	66 (4.71)	137 (9.79)	67 (4.79)
61	Oji C. Fractures of the facial skeleton in children: a survey of patients under the age of 11 years. J Craniomaxillofac Surg. 1998 Oct;26(5):322-5. https://doi.org/10.1016/s1010-5182(98)80062-0	65 (2.95)	140 (6.36)	73 (3.32)
62	Lalloo R. Risk factors for major injuries to the face and teeth. Dent Traumatol. 2003 Feb;19(1):12-4. https://doi.org/10.1034/j.1600-9657.2003.00139.x	63 (3.71)	109 (6.41)	63 (3.71)
63	Garon MW, Merkle A, Wright JT. Mouth protectors and oral trauma: a study of adolescent football players. J Am Dent Assoc. 1986 May;112(5):663-5.	63 (1.85)	137 (4.03)	83 (2.44)
64	Al-Asfour A, Andersson L, Al-Jame Q. School teachers’ knowledge of tooth avulsion and dental first aid before and after receiving information about avulsed teeth and replantation. Dent Traumatol. 2008 Feb;24(1):43-9. https://doi.org/10.1111/j.1600-9657.2006.00476.x	62 (5.17)	176 (14.67)	59 (4.92)
65	Cardoso M, de Carvalho Rocha MJ. Traumatized primary teeth in children assisted at the Federal University of Santa Catarina, Brazil. Dent Traumatol. 2002 Jun;18(3):129-33. https://doi.org/10.1034/j.1600-9657.2002.00030.x	62 (3.44)	156 (8.67)	71 (3.94)
66	Yamada T, Sawaki Y, Tomida S, Tohnai I, Ueda M. Oral injury and mouthguard usage by athletes in Japan. Endod Dent Traumatol. 1998 Apr;14(2):84-7. https://doi.org/10.1111/j.1600-9657.1998.tb00816.x	62 (2.82)	106 (4.82)	69 (3.14)
67	Petti S, Cairella G, Tarsitani G. Childhood obesity: a risk factor for traumatic injuries to anterior teeth. Endod Dent Traumatol. 1997 Dec;13(6):285-8. https://doi.org/10.1111/j.1600-9657.1997.tb00057.x	62 (2.70)	142 (6.17)	69 (3.00)
68	Fried I, Erickson P. Anterior tooth trauma in the primary dentition: incidence, classification, treatment methods, and sequelae: a review of the literature. ASDC J Dent Child. 1995 Jul-Aug;62(4):256-61.	62 (2.48)	127 (5.08)	73 (2.92)
69	Tanaka N, Uchide N, Suzuki K, Tashiro T, Tomitsuka K, Kimijima Y, Amagasa T. Maxillofacial fractures in children. J Craniomaxillofac Surg. 1993 Oct;21(7):289-93. https://doi.org/10.1016/s1010-5182(05)80349-x	62 (2.30)	130 (4.81)	59 (2.19)
70	Hunter ML, Hunter B, Kingdon A, Addy M, Dummer PM, Shaw WC. Traumatic injury to maxillary incisor teeth in a group of South Wales school children. Endod Dent Traumatol. 1990 Dec;6(6):260-4. https://doi.org/10.1111/j.1600-9657.1990.tb00429.x	62 (2.07)	126 (4.20)	78 (2.60)
71	Sgan-Cohen HD, Megnagi G, Jacobi Y. Dental trauma and its association with anatomic, behavioral, and social variables among fifth and sixth grade schoolchildren in Jerusalem. Community Dent Oral Epidemiol. 2005 Jun;33(3):174-80. https://doi.org/10.1111/j.1600-0528.2005.00202.x	61 (4.07)	115 (7.67)	65 (4.33)
72	Pohl Y, Filippi A, Kirschner H. Results after replantation of avulsed permanent teeth. I. Endodontic considerations. Dent Traumatol. 2005 Apr;21(2):80-92. https://doi.org/10.1111/j.1600-9657.2004.00297.x	61 (4.07)	149 (9.93)	71 (4.73)
73	Al-Jundi SH. Type of treatment, prognosis, and estimation of time spent to manage dental trauma in late presentation cases at a dental teaching hospital: a longitudinal and retrospective study. Dent Traumatol. 2004 Feb;20(1):1-5. https://doi.org/10.1111/j.1600-4469.2004.00218.x	61 (3.81)	129 (8.06)	69 (4.31)
74	Robertson A, Lundgren T, Andreasen JO, Dietz W, Hoyer I, Norén JG. Pulp calcifications in traumatized primary incisors. A morphological and inductive analysis study. Eur J Oral Sci. 1997 Jun;105(3):196-206.https://doi.org/10.1111/j.1600-0722.1997.tb00201.x	61 (2.65)	100 (4.35)	66 (2.87)
75	Pacheco LF, Filho PF, Letra A, Menezes R, Villoria GE, Ferreira SM. Evaluation of the knowledge of the treatment of avulsions in elementary school teachers in Rio de Janeiro, Brazil. Dent Traumatol. 2003 Apr;19(2):76-8. https://doi.org/10.1034/j.1600-9657.2003.00109.x	60 (3.53)	123 (7.24)	63 (3.71)
76	Kania MJ, Keeling SD, McGorray SP, Wheeler TT, King GJ. Risk factors associated with incisor injury in elementary school children. Angle Orthod. 1996;66(6):423-32. https://doi.org/10.1043/0003-3219(1996)066<0423:RFAWII>2.3.CO;2	60 (2.50)	110 (4.58)	60 (2.50)
77	O’Neil DW, Clark MV, Lowe JW, Harrington MS. Oral trauma in children: a hospital survey. Oral Surg Oral Med Oral Pathol. 1989 Dec;68(6):691-6. https://doi.org/10.1016/0030-4220(89)90157-6	60 (1.94)	129 (4.16)	74 (2.39)
78	Miller EK, Lee JY, Tawil PZ, Teixeira FB, Vann WF Jr. Emerging therapies for the management of traumatized immature permanent incisors. Pediatr Dent. 2012 Jan-Feb;34(1):66-9.	59 (7.38)	120 (15.00)	69 (8.63)
79	Al-Badri S, Kinirons M, Cole B, Welbury R. Factors affecting resorption in traumatically intruded permanent incisors in children. Dent Traumatol. 2002 Apr;18(2):73-6. https://doi.org/10.1034/j.1600-9657.2002.180205.x	59 (3.28)	103 (5.72)	70 (3.89)
80	Donaldson M, Kinirons MJ. Factors affecting the time of onset of resorption in avulsed and replanted incisor teeth in children. Dent Traumatol. 2001 Oct;17(5):205-9. https://doi.org/10.1034/j.1600-9657.2001.170503.x	59 (3.11)	191 (10.05)	84 (4.42)
81	Soriano EP, Caldas AF Jr, Góes PS. Risk factors related to traumatic dental injuries in Brazilian schoolchildren. Dent Traumatol. 2004 Oct;20(5):246-50. https://doi.org/10.1111/j.1600-9657.2004.00246.x	58 (3.63)	155 (9.69)	69 (4.31)
82	Jessee SA. Physical manifestations of child abuse to the head, face and mouth: a hospital survey. ASDC J Dent Child. 1995 Jul-Aug;62(4):245-9.	58 (2.32)	141 (5.64)	83 (3.32)
83	Al-Jundi SH. Dental emergencies presenting to a dental teaching hospital due to complications from traumatic dental injuries. Dent Traumatol. 2002 Aug;18(4):181-5. https://doi.org/10.1034/j.1600-9657.2002.02081.x	57 (3.17)	137 (7.61)	63 (3.50)
84	Güven O, Keskin A. Remodelling following condylar fractures in children. J Craniomaxillofac Surg. 2001 Aug;29(4):232-7. https://doi.org/10.1054/jcms.2001.0228	57 (3.00)	116 (6.11	56 (2.95)
85	Naidoo S, Sheiham A, Tsakos G. Traumatic dental injuries of permanent incisors in 11- to 13-year-old South African schoolchildren. Dent Traumatol. 2009 Apr;25(2):224-8. https://doi.org/10.1111/j.1600-9657.2008.00749.x	56 (5.09)	116 (10.55)	61 (5.55)
86	Skaare AB, Jacobsen I. Primary tooth injuries in Norwegian children (1-8 years). Dent Traumatol. 2005 Dec;21(6):315-9. https://doi.org/10.1111/j.1600-9657.2005.00362.x	56 (3.73)	167 (11.13)	65 (4.33)
87	Artun J, Behbehani F, Al-Jame B, Kerosuo H. Incisor trauma in an adolescent Arab population: prevalence, severity, and occlusal risk factors. Am J Orthod Dentofacial Orthop. 2005 Sep;128(3):347-52. https://doi.org/10.1016/j.ajodo.2004.06.032	56 (3.73)	129 (8.60)	63 (4.20)
88	Iatrou I, Theologie-Lygidakis N, Tzerbos F. Surgical protocols and outcome for the treatment of maxillofacial fractures in children: 9 years’ experience. J Craniomaxillofac Surg. 2010 Oct;38(7):511-6. https://doi.org/10.1016/j.jcms.2010.02.008	55 (5.50)	96 (9.60)	60 (6.00)
89	Thorén H, Iizuka T, Hallikainen D, Nurminen M, Lindqvist C. An epidemiological study of patterns of condylar fractures in children. Br J Oral Maxillofac Surg. 1997 Oct;35(5):306-11. https://doi.org/10.1016/s0266-4356(97)90401-0	53 (2.30)	105 (4.57)	52 (2.26)
90	Nikoui M, Kenny DJ, Barrett EJ. Clinical outcomes for permanent incisor luxations in a pediatric population. III. Lateral luxations. Dent Traumatol. 2003 Oct;19(5):280-5. https://doi.org/10.1034/j.1600-9657.2003.00209.x	52 (3.06)	120 (7.06)	59 (3.47)
91	Hamilton FA, Hill FJ, Holloway PJ. An investigation of dento-alveolar trauma and its treatment in an adolescent population. Part 2: Dentists’ knowledge of management methods and their perceptions of barriers to providing care. Br Dent J. 1997 Feb 22;182(4):129-33. https://doi.org/10.1038/sj.bdj.4809323	52 (2.26)	114 (4.96)	62 (2.70)
92	Perez R, Berkowitz R, McIlveen L, Forrester D. Dental trauma in children: a survey. Endod Dent Traumatol. 1991 Oct;7(5):212-3. https://doi.org/10.1111/j.1600-9657.1991.tb00438.x	52 (1.79)	144 (4.97)	65 (2.24)
93	Thorén H, Hallikainen D, Iizuka T, Lindqvist C. Condylar process fractures in children: a follow-up study of fractures with total dislocation of the condyle from the glenoid fossa. J Oral Maxillofac Surg. 2001 Jul;59(7):768-73; discussion 773-4. https://doi.org/10.1053/joms.2001.23369	51 (2.68)	118 (6.21)	57 (3.00)
94	Díaz JA, Bustos L, Brandt AC, Fernández BE. Dental injuries among children and adolescents aged 1-15 years attending to public hospital in Temuco, Chile. Dent Traumatol. 2010 Jun;26(3):254-61. https://doi.org/10.1111/j.1600-9657.2010.00878.x	50 (5.00)	149 (14.90)	60 (6.00)
95	Gerbino G, Roccia F, Bianchi FA, Zavattero E. Surgical management of orbital trapdoor fracture in a pediatric population. J Oral Maxillofac Surg. 2010 Jun;68(6):1310-6. https://doi.org/10.1016/j.joms.2009.12.037	50 (5.00)	89 (8.90)	50 (5.00)
96	Navabazam A, Farahani SS. Prevalence of traumatic injuries to maxillary permanent teeth in 9- to 14-year-old school children in Yazd, Iran. Dent Traumatol. 2010 Apr;26(2):154-7. https://doi.org/10.1111/j.1600-9657.2009.00861.x	50 (5.00)	148 (14.80)	58 (5.80)
97	Choi J, Oh N, Kim IK. A follow-up study of condyle fracture in children. Int J Oral Maxillofac Surg. 2005 Dec;34(8):851-8. https://doi.org/10.1016/j.ijom.2005.04.005	50 (3.33)	105 (7.00)	53 (3.53)
98	Yerit KC, Hainich S, Enislidis G, Turhani D, Klug C, Wittwer G, Ockher M, Undt G, Kermer C, Watzinger F, Ewers R. Biodegradable fixation of mandibular fractures in children: stability and early results. Oral Surg Oral Med Oral Pathol Oral Radiol Endod. 2005 Jul;100(1):17-24. https://doi.org/10.1016/j.tripleo.2004.11.013	50 (3.33)	103 (6.87)	48 (3.20)
99	Zuhal K, Semra OE, Hüseyin K. Traumatic injuries of the permanent incisors in children in southern Turkey: a retrospective study. Dent Traumatol. 2005 Feb;21(1):20-5. https://doi.org/10.1111/j.1600-9657.2004.00265.x	50 (3.33)	168 (11.20)	61 (4.07)
100	Altun C, Cehreli ZC, Güven G, Acikel C. Traumatic intrusion of primary teeth and its effects on the permanent successors: a clinical follow-up study. Oral Surg Oral Med Oral Pathol Oral Radiol Endod. 2009 Apr;107(4):493-8. https://doi.org/10.1016/j.tripleo.2008.10.016	49 (4.45)	108 (9.82)	50 (4.55)

The 100 most cited articles were cited 7,932 times in WoS-CC, ranging from 49 to 176 citations. Twenty articles had at least 100 citations and were considered highly cited. Self-citations accounted for 4.76% of the total number of citations and were considered in the study. Citations were higher in Google Scholar (n = 17,901 citations) and Scopus (n = 8,894 citations). Strong positive correlations were found in the number of citations between WoS-CC and Google Scholar (r = 0.929; p < 0.001), between WoS-CC and Scopus (r = 0.976; p < 0.001), and between Google Scholar and Scopus (r = 0.903; p < 0.001). The most cited article was “Pediatric facial fractures - evolving patterns of treatment”, which was a cohort study by Posnick, Wells and Pron (1993) published in Journal of Oral and Maxillofacial Surgery, with a total of 176 citations in WoS-CC (Table 1).

**Figure 1:**
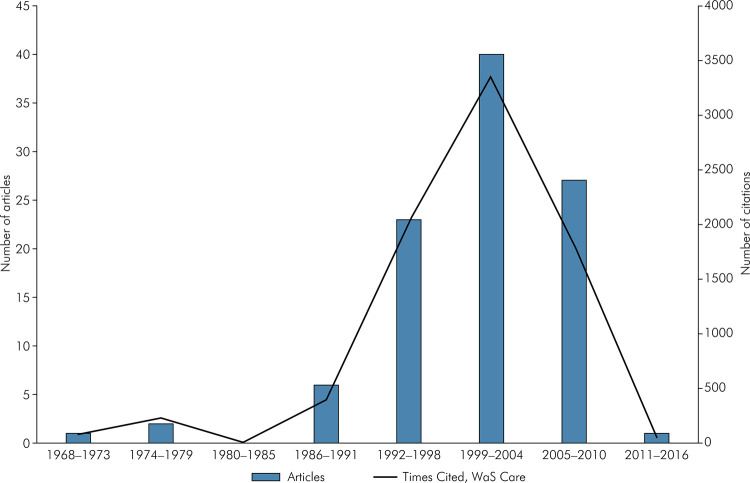
Number of citations and publications over time.

The year of publication ranged from 1968 to 2012 (Figure 1). The year 2001 had the largest number of published articles (n = 14). The oldest article was an integrative review by Rowe NL which was published in 1968 and cited 81 times in WoS-CC. The most recent article was a cross-sectional study by Miller EK et al. (2012), which was cited 59 times in WoS-CC (Table1).

The analysis of the types of orofacial trauma revealed that traumatic dental injury was the most addressed subject amongst the 100 most cited articles about orofacial trauma (n = 83; 6,616 citations). The age of the participants ranged from 0 to 19 years; 53 studies included preschoolers, schoolchildren, and/or adolescents combined. Most of the studies on traumatic dental injury addressed exclusively permanent dentition (50 articles; 4,029 citations).

**Figure 2:**
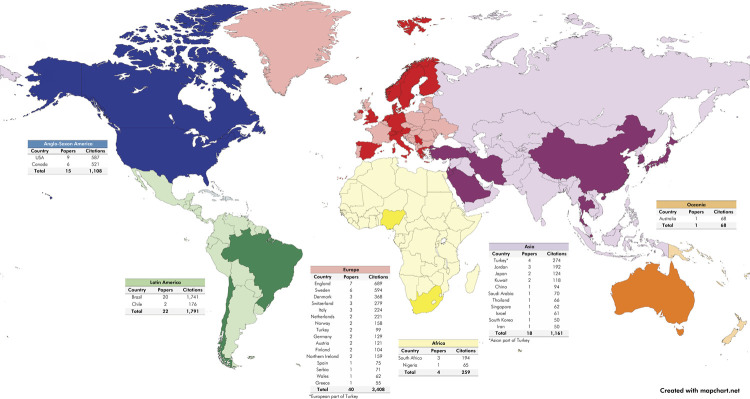
Worldwide distribution of the 100 most cited articles about orofacial trauma in children and adolescents.

The continents with the largest number of articles were Europe (n = 40 articles; 3,408 citations), Latin America (n = 22 articles; 1,917 citations), and North America (n = 15 articles; 1,108 citations) (Figure 2). The country with the largest contribution was Brazil (20 articles; 1,741 citations), followed by the United States of America (9 articles; 587 citations), and England (7 articles; 689 citations) (Figure 2). The institution with the most articles on the top 100 list was *Universidade do Sul de Santa Catarina*/Brazil (5 articles; 492 citations), followed by University College London/England (3 articles; 338 citations), *Universidade Federal de Minas Gerais/*Brazil (3 articles; 275 citations), and *Universidade Federal de Santa* Catarina/Brazil (3 articles; 231 citations).

**Figure 3:**
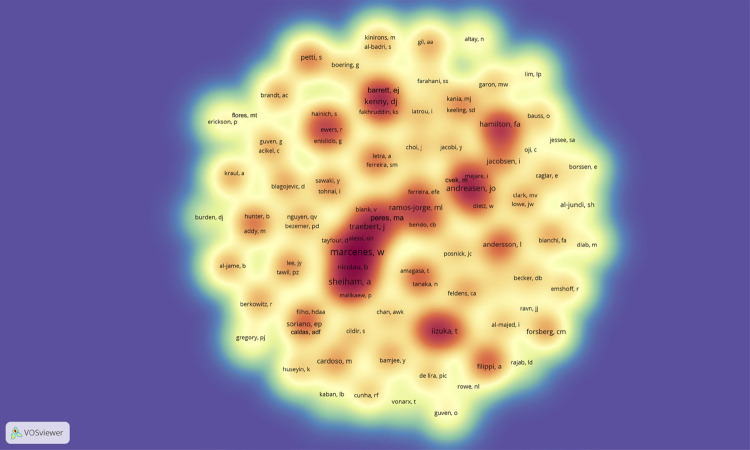
VOSviewer co-authorship density map demonstrating the clusters of authors of the 100 most cited articles about orofacial trauma in children and adolescents.

The author with the largest number of articles was Marcenes W (8 articles; 968 citations), followed by Sheiham A (5 articles; 512 citations), Traebert J (4 articles; 419 citations), Andreasen JO (4 articles; 373 citations), and Kenny DJ (4 articles; 272 citations). These authors worked mainly on five topics: etiology, prevalence, diagnosis, prevention, and treatment. The bibliometric networks detailed co-authorship relationships among authors of the 100 most cited articles. The main cluster contained prominent authors, such as Marcenes W and Sheiham A. Three other clusters were found with prominent authors: a) Peres MA and Ramos-Jorge ML; b) Kenny DJ and Barret EJ; c) Andreasens JO and Cvek M. National and international collaborations amongst authors were also found (Figure 3).

**Figure 4:**
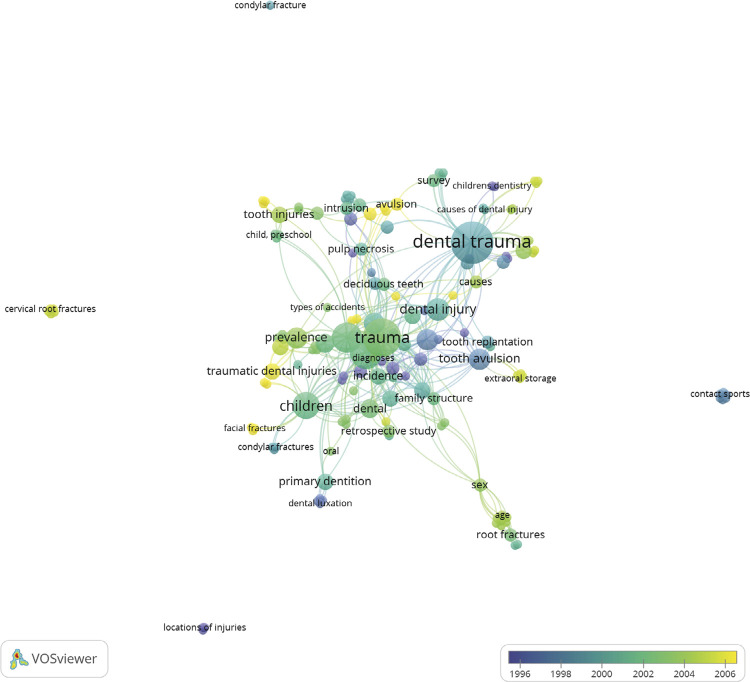
Author keywords by decade.

The most common study design was cross-sectional (50 articles; 3,978 citations) (Table 2). There was only one systematic review, but it stood out due to the high citation ratio (135.0). A total of 152 keywords were identified. The most frequent of which were “Trauma”, “Dental Trauma”, and “Children”. Figure 4 presents an overlay map showing the most frequent terms by decade. In the 1990s, the most frequent terms were “tooth avulsion”, “tooth reimplantation”, “pulp necrosis”, “dental luxation”, and “children’s dentistry”. Beginning in the year 2006, the most frequent terms were “avulsion”, “traumatic dental injuries”, and “facial fractures”.

**Table 2:** Frequencies of characteristics of the 100 most cited articles on orofacial trauma in children and adolescents.

Study design	Number of articles	Number of citations	Citation ratio
Cross-sectional	50	17,784	355.7
Cohort	37	12,613	340.9
Literature review	4	1,621	405.3
Case-control	2	604	302
Case report	2	259	259
Systematic review	1	574	574
Case report and literature review	1	410	410
Integrative review	1	353	353
Case series	1	281	281
In vitro study	1	227	227

Citation ratio: number of citations to number of articles in each study design and subject.

The most commonly used criteria for traumatic dental injury diagnosis were the criteria proposed by Andreasen^21-27^ (18 articles; 1,505 citations), whereas the most commonly used criteria for orofacial trauma diagnosis were the criteria proposed by Le Fort^28^(3 articles; 260 citations). Twenty-two articles did not report the index used for the evaluation of orofacial trauma. Among these articles, 19 of them addressed traumatic dental injuries and three addressed facial trauma. The most frequent field was epidemiology (73 articles; 5,791 citations). Dental Traumatology was the journal with the largest contribution (Table 3).

**Table 3:** Journal of publication of the 100 most cited articles on oral traumatology in children and adolescents.

Revistas	Numbers of articles	Numbers of citations	Citation ratio
Dental Traumatology Total	58	20,539	354.1
Dental Traumatology	48	16,597	345.8
Endodontics & Dental Traumatology	10	3,942	394.2
Journal of Oral and Maxillofacial Surgery	5	1,924	384.8
Journal of Cranio-Maxillofacial Surgery	4	969	242.3
Swedish Dental Journal	3	1,451	483.7
Community Dentistry and Oral Epidemiology	3	1,069	356.3
International Journal of Oral and Maxillofacial Surgery	3	800	266.7
British Dental Journal	2	888	444
International Dental Journal	1	588	588
European Journal of Orthodontics	2	978	489
Journal of the American Dental Association	2	714	357
Australian Dental Journal	2	712	356
British Journal of Oral & Maxillofacial Surgery	2	545	272.5
Journal of Dentistry For Children	2	544	272
Oral Surgery Oral Medicine Oral Pathology Oral Radiology and Endodontics	2	464	232
Operative Dentistry	1	430	430
Journal of Oral Surgery	1	353	353
Quintessence International	1	328	328
Community Dental Health	1	270	270
American Journal of Orthodontics And Dentofacial Orthopedics	1	248	248
Pediatric Dentistry	1	248	248
Angle Orthodontist	1	230	230
European Journal of Oral Sciences	1	227	227
Oral Surgery Oral Medicine Oral Pathology Oral Radiology and Endodontology	1	207	207

## Discussion

The present study identified the 100 most cited articles in the field of orofacial trauma in children and adolescents and performed qualitative-quantitative analyses of these articles. Bibliometric analyses are relevant, as the number of citations of an article can represent its impact on clinical practice and the development of future studies.^5,29-31^

In the literature, an article is considered highly cited when the number of citations is greater than 400.^18,32^ For specific topics, however, such as oral traumatology, 100 citations is sufficient for an article to be considered highly cited in the field.^13,33,34^ Among the articles included in this study, 20 had at least 100 citations, demonstrating considerable influence in clinical practice and guiding other studies.^18,33^

The WoS was chosen as the reference database for the present study because it allows retrieving publications as far back as 1945. This database includes peer-reviewed articles published in periodicals throughout the world.^31,35^ In contrast, the Scopus database has the limitation of containing only articles published since 1996, which can lead to bias when selecting the most cited articles.^11,34,35^ The large number of citations in the Google Scholar database was due to the fact that, unlike WoS and Scopus, this database contains documents that have not been peer reviewed and whose scientific value can therefore not be guaranteed. Despite the difference in the number of citations, strong positive correlations were found between the different databases. Thus, as the number of citations of the articles in the WoS database increased, this also tended to be the case in Google Scholar and Scopus.^34^

The highly cited articles in this bibliometric analysis were published from 1974 to 2005. The most cited study addressed maxillofacial trauma and was written by Posnick, Wells and Pron (Table 1). The wide scope of the data reported in this retrospective cohort study may explain the greater number of citations. The other highly cited studies addressed important topics, such as epidemiology, incidence, prevalence, and associated factors of orofacial trauma. The most recent highly cited article was written by de Pohl Y et al. (Table 1). The follow-up time greater than one year and the innovation of a promising method for the treatment of complex traumatic dental injuries may explain the number of citations of this study.

The article by Miller EK et al. was the most recent among the 100 most cited articles (Table1). In 2012, pulp revascularization was the main focus of studies. Miller EK et al., after finding satisfactory results, suggested a new treatment protocol for these cases. This shows that, although articles accumulate citations over time, theoretically favoring older articles, novel information can also contribute to an increase in citation density of more recent studies.^36,37^

Europe, South America, and North America were the continents with the most articles on the list of the 100 most cited. Prior to the 2000s, the major focus of studies, especially in the field of pediatric dentistry, was cariology. However, interest in traumatic dental injuries emerged in the scientific community in the beginning of the 2000s, especially after the studies developed by Traebert and Moreira (Table 1).^38-41^ Brazil was the country with the highest number of articles, which may be explained by the fact that the prevalence of traumatic dental injury is high in the country.^39,42^ The United States of America were the second country with the most articles, which may be due to the fact that the largest research centers in the world are located in the country.^42^ Moreover, due to investments in the funding of research and infrastructure, developed countries have more consolidated health research centers,^43-45^ which is in agreement with data from other bibliometric studies in denstistry.^13,19,37^

Five authors were the most cited among the articles included in this bibliometric analysis: Marcenes, Sheiham, Traebert, Andreasen and Kenny (Table 1). These authors worked mainly on five topics: etiology, prevalence, diagnosis, prevention, and treatment, demonstrating their important contributions to the study, understanding, and management of orofacial trauma. In the bibliometric study by Kramer et al.,^14^ etiology and treatment were also among the most discussed topics. Traebert developed a set of pioneering studies related to trauma in the permanent dentition beginning in the year 2000 (Table 1). In co-authorship with Marcenes W, the researcher developed pioneering cross-sectional studies on prevalence, treatment needs, and factors associated with trauma in permanent dentition. These studies were subsequently cited by most studies on the subject. Kenny published several literature reviews and guidelines on the treatment of traumatic dental injuries (Table 1). Andreasen JO may be considered the major scholar on the subject.^34^ His first publication available in PubMed dates back to 1967. In partnership with Andreasen FM, the researcher developed important histological studies that provided the basis for treatment protocols and guidelines for primary and permanent dentitions. Previous studies have identified Andreassen as the most productive author in the field of oral traumatology.^13,33^ He was also one of those who took the initiative to found the IADT, which has promoted 21 conferences on dental traumatology throughout the world and serves as a guide for the management of orofacial trauma.^34^Moreover, national and international collaborations are found among the authors of the articles. The existence of these working groups focused on particular subjects, consequently increases the frequency of self-citations, especially considering the fact that the topic is more restricted.^18^

The cross-sectional design was the most frequently used among the studies, as well as in the bibliometric study by Kramer et al.^14^ This design may have been the most frequent because such studies are efficient, fast and inexpensive, making this design more common in dental studies to evaluate prevalence in large populations and raise etiological hypotheses.^46,47^ Only one systematic review was among the 100 most cited articles. Clinical practice in orofacial trauma has not been based on high-quality clinical evidence.^45^ Well-designed clinical trials are needed for the application of better scientific evidence in the treatment of orofacial trauma.

The Andreassen and Le Fort criteria were the most used and are considered classic criteria for the evaluation of traumatic dental injuries and maxillofacial trauma, respectively. Among the studies that evaluated the occurrence of orofacial trauma, 22 failed to report the index used for diagnosis. This hinders comparisons among studies and can also have repercussions regarding the level of agreement in diagnosis among evaluators, putting into question the validity of studies.

Most studies addressed the preschool, school or adolescent age groups combined. By studying a wider age range, studies are able to present the characteristics of orofacial trauma at different ages. However, future studies focusing on each age group are needed to enable more assertive prevention and treatment guidelines for orofacial trauma. One limitation of the current study is that the research was conducted in 2021, and the results have since been updated; therefore, we encourage further studies.

## Conclusion

This quantitative and qualitative bibliometric analysis can be useful for clinicians, researchers, and public policy development. The 100 most cited articles were mainly cross-sectional epidemiology studies and that used Andreasen criteria and were published by Brazilian authors. The findings demonstrated the need for studies focused on improving the treatment of orofacial trauma.

## References

[1] Flores MT (2002). Traumatic injuries in the primary dentition. Dent Traumatol.

[2] Petti S, Glendor U, Andersson L (2018). World traumatic dental injury prevalence and incidence, a meta-analysis-One billion living people have had traumatic dental injuries. Dent Traumatol.

[3] Almasri M (2013). Severity and causality of maxillofacial trauma in the Southern region of Saudi Arabia. Saudi Dent J.

[4] Feldens CA, Kramer PF, Ferreira SH, Spiguel MH, Marquezan M (2010). Exploring factors associated with traumatic dental injuries in preschool children: a Poisson regression analysis. Dent Traumatol.

[5] Piccininni P, Clough A, Padilla R, Piccininni G (2017). Dental and orofacial injuries. Clin Sports Med.

[6] Yang X, Sun W, Wang Z, Ji AP, Bai J (2021). [Clinical analysis of children and adolescents emergency dental trauma cases]. Beijing Da Xue Xue Bao.

[7] Zaror C, Martínez-Zapata MJ, Abarca J, Díaz J, Pardo Y, Pont À (2018). Impact of traumatic dental injuries on quality of life in preschoolers and schoolchildren: A systematic review and meta-analysis. Community Dent Oral Epidemiol.

[8] Lopez D, Waidyatillake N, Zaror C, Mariño R (2019). Impact of uncomplicated traumatic dental injuries on the quality of life of children and adolescents: a systematic review and meta-analysis. BMC Oral Health.

[9] Ellegaard O, Wallin JA (2015). The bibliometric analysis of scholarly production: how great is the impact?. Scientometrics.

[10] Moed HF (2009). New developments in the use of citation analysis in research evaluation. Arch Immunol Ther Exp (Warsz).

[11] Guo J, Gu D, Zhao T, Zhao Z, Xiong Y, Sun M (2021). Trends in Piezo channel research over the past decade: a bibliometric analysis. Front Pharmacol.

[12] Mattos FF, Perazzo MF, Vargas-Ferreira F, Martins-Júnior PA, Paiva SM (2021). Top 100 most-cited papers in core dental public health journals: bibliometric analysis. Community Dent Oral Epidemiol.

[13] Jafarzadeh H, Shirazi AS, Andersson L (2015). The most-cited articles in dental, oral, and maxillofacial traumatology during 64 years. Dent Traumatol.

[14] Kramer PF, Onetto J, Flores MT, Borges TS, Feldens CA (2016). Traumatic dental injuries in the primary dentition: a 15-year bibliometric analysis of Dental Traumatology. Dent Traumatol.

[15] Tahim A, Patel K, Bridle C, Holmes S (2016). The 100 most cited articles in facial trauma: a bibliometric analysis. J Oral Maxillofac Surg.

[16] Liu F, Wu TT, Lei G, Fadlelseed AF, Xie N, Wang DY (2020). Worldwide tendency and perspectives in traumatic dental injuries: a bibliometric analysis over two decades (1999-2018). Dent Traumatol.

[17] Cochrane Glossary https://epoc.cochrane.org/sites/epoc.cochrane.org/files/public/uploads/SURE-Guides-v2.1/Collectedfiles/source/glossary.html.

[18] Garfield E What is a citation classic?.

[19] Perazzo MF, Otoni AL, Costa MS, Granville-Granville AF, Paiva SM, Martins-Júnior PA (2019). The top 100 most-cited papers in Paediatric Dentistry journals: A bibliometric analysis. Int J Paediatr Dent.

[20] van Eck NJ, Waltman L (2010). Software survey: VOSviewer, a computer program for bibliometric mapping. Scientometrics.

[21] Andreasen JO (1990). Atlas of replantation and transplantation of teeth.

[22] Andreasen FM, Andreasen JO (1985). Diagnosis of luxation injuries: the importance of standardized clinical, radiographic and photographic techniques in clinical investigations. Endod Dent Traumatol.

[23] Andreasen JO, Andreasen FM (1990). Essentials of traumatic injuries to the teeth.

[24] Andreasen J (1981). Traumatic injuries of the teeth.

[25] Andreasen FM, Andreasen JO (1995). Treatment strategy for root fractures in the permanent dentition. Aust Endod Newsl.

[26] Andreasen JO, Andreasen FM (1993). Textbook and color atlas of traumatic injuries to the teeth.

[27] Andreasen JO, Andreasen FM, Andersson L (2007). Textbook and color atlas of traumatic injuries to the teeth.

[28] Le Fort R. (1901). Étude expérimentale sur les fractures de la mâchoire supérieure. Rev Chir. Paris..

[29] Shekhani HN, Shariff S, Bhulani N, Khosa F, Hanna TN (2017). Bibliometric analysis of manuscript characteristics that influence citations: A comparison of six major radiology journals. AJR Am J Roentgenol.

[30] Eyre-Walker A, Stoletzki N, Eyre-WalkerA (2013). The assessment of science: the relative merits of post-publication review, the impact factor, and the number of citations. PLoS Biol.

[31] Kulkarni AV, Aziz B, Shams I, Busse JW (2009). Comparisons of citations in Web of Science, Scopus, and Google Scholar for articles published in general medical journals. JAMA.

[32] Van Noorden R, Maher B, Nuzzo R (2014). The top 100 papers. Nature.

[33] Ahmad P, Vincent Abbott P, Khursheed Alam M, Ahmed Asif J (2020). A bibliometric analysis of the top 50 most cited articles published in the Dental Traumatology. Dent Traumatol.

[34] Santos PS, Santos N, Moccelini BS, Bolan M, Santana CM, Martins-Junior PA (2021). The top 100 most-cited papers authored by Dr. Jens Ove Andreasen: a bibliometric analysis. Dent Traumatol.

[35] Bakkalbasi N, Bauer K, Glover J, Wang L (2006). Three options for citation tracking: Google Scholar, Scopus and Web of Science. Biomed Digit Libr.

[36] Feijoo JF, Limeres J, Fernández-Varela M, Ramos I, Diz P (2014). The 100 most cited articles in dentistry. Clin Oral Investig.

[37] Baldiotti AL, Amaral-Freitas G, Barcelos JF, Freire-Maia J, Perazzo MF, Freire-Maia FB (2021). The Top 100 Most-Cited Papers in Cariology: A Bibliometric Analysis. Caries Res.

[38] Traebert J, Moreira EA (2001). Behavioral eating disorders and their effects on the oral health in adolescence. Pesqui Odontol Bras.

[39] Marcenes W, Zabot NE, Traebert J (2001). Socio-economic correlates of traumatic injuries to the permanent incisors in schoolchildren aged 12 years in Blumenau, Brazil. Dent Traumatol.

[40] Traebert JL, Peres MA, Galesso ER, Zabot NE, Marcenes W (2001). Prevalence and severity of dental caries among schoolchildren aged six and twelve. Rev Saude Publica.

[41] Peres MA, Traebert J, Marcenes W (2001). Calibration of examiners for dental caries epidemiologic studies. Cad Saude Publica.

[42] Shadgan B, Roig M, Hajghanbari B, Reid WD (2010). Top-cited articles in rehabilitation. Arch Phys Med Rehabil.

[43] Gil-Montoya JA, Navarrete-Cortes J, Pulgar R, Santa S, Moya-Anegón F (2006). World dental research production: an ISI database approach (1999-2003). Eur J Oral Sci.

[44] Benzer A, Pomaroli A, Hauffe H, Schmutzhard E (1993). Geographical analysis of medical publications in 1990. Lancet.

[45] Feldens CA, Kramer PF, Feldens EG (2013). Exploring the profile of articles on traumatic dental injuries in pediatric dental journals. Dent Traumatol.

[46] Levin KA (2006). Study design III: cross-sectional studies. Evid Based Dent.

[47] Torriani DD, Ferro RL, Bonow ML, Santos IS, Matijasevich A, Barros AJ (2014). Dental caries is associated with dental fear in childhood: findings from a birth cohort study. Caries Res.

